# Disease-Specific Clinical Correlates of Vitamin D in Systemic Lupus Erythematosus and Behçet’s Disease

**DOI:** 10.3390/medicina62101822

**Published:** 2026-09-22

**Authors:** Mustafa Gür, Ahmet Karataş, Burak Öz, Sümeyye Şahin, Yusuf Doğan, Nevzat Gözel, Süleyman Serdar Koca

**Affiliations:** 1 Department of Rheumatology, Faculty of Medicine, Fırat University, 23119 Elazığ, Turkey; drakaratas@yahoo.com (A.K.); boz@firat.edu.tr (B.Ö.); sumeyye_balta@hotmail.com (S.Ş.); yusuf.230@hotmail.com (Y.D.); kocassk@yahoo.com (S.S.K.); 2 Department of Internal Medicine, Faculty of Medicine, Fırat University, 23119 Elazığ, Turkey; drngozel@hotmail.com

**Keywords:** vitamin D, systemic lupus erythematosus, Behçet’s disease, iron metabolism, metabolic syndrome

## Abstract

*Background and Objectives*: Vitamin D deficiency is common in systemic lupus erythematosus (SLE) and Behçet’s disease (BD). However, whether it is associated with the same clinical parameters in both conditions remains unknown. Our objective is to compare the clinical correlates of serum 25-hydroxyvitamin D [25(OH)D] levels in patients with SLE and BD. *Materials and Methods*: This exploratory cross-sectional study included 38 patients with SLE and 32 with BD. Spearman’s correlation analyses, partial correlations controlling for sex, and tertile analyses were performed separately for each disease group. *Results:* Vitamin D deficiency [25(OH)D < 20 ng/mL] was similarly prevalent in SLE (64.9%) and BD (62.5%). However, distinct disease-specific correlation profiles were noted. In SLE, serum 25(OH)D levels were inversely correlated with body mass index (BMI) (*r* = −0.47, *p* = 0.003) and parathyroid hormone (PTH) (*r* = −0.40, *p* = 0.01), with no significant associations with inflammatory or haematological parameters. In patients with BD, 25(OH)D levels were positively correlated with haemoglobin (*r* = 0.63, *p* < 0.001), haematocrit (*r* = 0.58, *p* < 0.001), ferritin (*r* = 0.47, *p* = 0.008), uric acid (*r* = 0.48, *p* = 0.005), transferrin saturation (*r* = 0.45, *p* = 0.02), and triglycerides (*r* = 0.44, *p* = 0.01), while they were inversely correlated with total iron-binding capacity (*r* = −0.50, *p* = 0.01) and erythrocyte sedimentation rate (ESR) (*r* = −0.44, *p* = 0.01). After adjusting for sex, the association between 25(OH)D and haemoglobin remained significant (partial *r* = 0.43, *p* = 0.01), as did the associations with haematocrit (partial *r* = 0.42, *p* = 0.02) and triglycerides (partial *r* = 0.43, *p* = 0.02). In contrast, the correlations with ferritin, ESR, uric acid, and transferrin saturation were attenuated and no longer statistically significant. Tertile analyses demonstrated dose–response relationships between vitamin D levels and BMI (*p* = 0.01) and PTH (*p* = 0.008) in SLE, and between vitamin D levels and haemoglobin (*p* = 0.001) and ferritin (*p* = 0.02) in BD. *Conclusions*: Despite the similar prevalence of vitamin D deficiency, patients with SLE and BD exhibit distinct vitamin D-associated clinical profiles. Vitamin D status was primarily associated with metabolic parameters in SLE and haematological parameters in BD. These disease-specific patterns may reflect differences in the underlying inflammatory pathways and pathophysiological mechanisms involved.

## 1. Introduction

Vitamin D deficiency is highly prevalent in autoimmune and inflammatory rheumatic diseases [1,2]. Serum 25-hydroxyvitamin D [25(OH)D] levels are consistently lower in patients with systemic lupus erythematosus (SLE) and Behçet’s disease (BD) compared with healthy populations, with deficiency rates exceeding 60% in both conditions [3,4,5,6]. Beyond its classical role in calcium–phosphorus homeostasis, vitamin D exerts immunomodulatory, anti-inflammatory, and metabolic effects through the vitamin D receptor expressed on immune cells, adipocytes, and vascular endothelium [2,7,8].

In SLE, vitamin D deficiency has been associated with higher disease activity, fatigue, metabolic syndrome, and cardiovascular risk [6,9,10]. SLE patients face particular risk due to photosensitivity-related sun avoidance, nephropathy-associated vitamin D metabolism impairment, and corticosteroid use [6]. In BD, a systemic vasculitis characterised by mucocutaneous, ocular, and vascular manifestations, vitamin D deficiency has been linked to disease activity and inflammatory markers [4,5,11]. However, few studies have directly compared the clinical correlates of vitamin D deficiency between SLE and BD, despite their distinct and complex pathogenic mechanisms. SLE is characterised by dysregulated innate and adaptive immune responses, including type I interferon signalling and autoantibody production, arising from interactions between genetic susceptibility and environmental triggers [12,13]. In contrast, BD is a systemic inflammatory vasculitis in which genetic susceptibility, environmental factors, neutrophil hyperreactivity, and dysregulated innate and adaptive immune responses contribute to disease development [14,15,16].

This distinction is clinically relevant because the same laboratory finding, low 25(OH)D, may be associated with different clinical consequences depending on the underlying disease. Identifying disease-specific patterns could inform targeted supplementation strategies and improve the interpretation of vitamin D levels in clinical practice.

This exploratory study aimed to compare the clinical correlates of vitamin D status in SLE and BD patients, with a focus on metabolic, inflammatory, and haematological parameters.

## 2. Materials and Methods

### 2.1. Study Design and Population

This single-centre cross-sectional study was conducted at the Rheumatology Clinic of Fırat University Hospital, Elazığ, Turkey. Thirty-eight patients with SLE (2019 EULAR/ACR criteria) and thirty-two patients with BD (International Study Group criteria) were enrolled consecutively. All patients were aged ≥18 years. Patients receiving vitamin D supplementation within the previous three months and those with chronic kidney disease stage ≥ 3, chronic liver disease, pregnancy, active malignancy, acute infection, or disorders affecting vitamin D metabolism were excluded. Written informed consent was obtained. The study was approved by the Fırat University Non-Interventional Research Ethics Committee and conducted in accordance with the Declaration of Helsinki.

### 2.2. Clinical and Laboratory Assessment

Demographics and disease duration were recorded. Fasting blood samples were analysed for 25(OH)D, parathyroid hormone (PTH), calcium, phosphorus, alkaline phosphatase (ALP), complete blood count (haemoglobin, haematocrit), iron parameters (serum iron, total iron-binding capacity [TIBC], ferritin, transferrin saturation [TSAT] calculated as serum iron/TIBC × 100), fasting glucose, fasting insulin, lipid profile (total cholesterol, triglycerides, low-density lipoprotein [LDL], high-density lipoprotein [HDL]), uric acid, albumin, C-reactive protein (CRP) (latex-enhanced immunoturbidimetric assay), erythrocyte sedimentation rate (ESR), glomerular filtration rate (GFR) and complement (C3 and C4, in SLE only). Serum 25(OH)D concentrations were measured using liquid chromatography–tandem mass spectrometry (LC-MS/MS) with a SCIEX QTRAP 4500 mass spectrometer (SCIEX, Framingham, MA, USA). The analytical method was calibrated using appropriate calibration standards and was validated for analytical precision and accuracy according to the laboratory’s established quality-control procedures. Homeostatic model assessment of insulin resistance (HOMA-IR) was calculated as fasting insulin × fasting glucose/405. Vitamin D status was categorised as deficient (<20 ng/mL), insufficient (20–29.9 ng/mL), or sufficient (≥30 ng/mL). Blood samples were collected consecutively during routine outpatient visits throughout the study period, irrespective of season. Seasonal variation in serum 25(OH)D levels was not controlled for.

### 2.3. Statistical Analysis

Analyses were performed using IBM SPSS Statistics for Windows, version 26.0 (IBM Corp., Armonk, NY, USA). Given the exploratory design of the study, no formal a priori sample size calculation was performed. Normality was assessed via the Shapiro–Wilk test. Continuous variables are reported as mean ± standard deviation (SD) or median (interquartile range [IQR]) as appropriate. Between-group comparisons used Student’s *t*-test or Mann–Whitney U test. Categorical variables were compared using chi-square or Fisher’s exact test. Within each disease group, Spearman rank correlations assessed associations between 25(OH)D and clinical parameters. Because the BD group had equal proportions of males and females, and because sex influences both vitamin D levels and haemoglobin concentrations, partial Spearman correlations controlling for sex were calculated for BD vitamin D associations. Sex-stratified subgroup analyses were also performed within the BD group. Patients were stratified into disease-specific 25(OH)D tertiles and compared using Kruskal–Wallis tests. Given the modest sample sizes and multiple comparisons tested without formal multiplicity correction, all findings should be interpreted as exploratory and hypothesis-generating. A two-tailed *p* < 0.05 was considered statistically significant.

## 3. Results

### 3.1. Patient Characteristics

Thirty-eight SLE and thirty-two BD patients were included. Groups were comparable in mean age (39.3 ± 12.1 vs. 40.4 ± 11.7 years, *p* = 0.70), body mass index (BMI) (24.6 vs. 24.1 kg/m^2^, *p* = 0.73), and disease duration (90 vs. 114 months, *p* = 0.33). The SLE group had a higher proportion of females (86.8% vs. 50.0%). SLE patients had lower serum iron (46.0 vs. 75.0 µg/dL, *p* = 0.02), lower TSAT (13.9% vs. 20.7%, *p* = 0.048), lower haemoglobin (12.7 ± 1.7 vs. 13.9 ± 1.9 g/dL, *p* = 0.007), higher ESR (23 vs. 11.5 mm/h, *p* = 0.03), and lower albumin (4.2 vs. 4.5 g/dL, *p* = 0.002) (Table 1).

### 3.2. Vitamin D Status

Vitamin D deficiency was highly prevalent and comparable between groups. In SLE, 64.9% were deficient (<20 ng/mL), 13.5% insufficient, and 21.6% sufficient (≥30 ng/mL). In BD, 62.5% were deficient, 28.1% insufficient, and 9.4% sufficient. Median 25(OH)D did not differ between groups (16.2 vs. 13.9 ng/mL, *p* = 0.37).

### 3.3. Disease-Specific Vitamin D Correlations

Despite similar vitamin D levels, different correlation patterns were observed between SLE and BD (Table 2).

In SLE, 25(OH)D correlated inversely with BMI (*r* = −0.47, *p* = 0.003) and PTH (*r* = −0.40, *p* = 0.01). No significant correlations were observed with haemoglobin, iron parameters, inflammatory markers, or lipids.

In BD, a different correlation pattern was observed, with significant associations involving haematological and inflammatory. Positive correlations were observed with haemoglobin (*r* = 0.63, *p* < 0.001), haematocrit (*r* = 0.58, *p* < 0.001), uric acid (*r* = 0.48, *p* = 0.005), ferritin (*r* = 0.47, *p* = 0.008), TSAT (*r* = 0.45, *p* = 0.02), and triglycerides (*r* = 0.44, *p* = 0.01). An inverse correlation was observed with ESR (*r* = −0.44, *p* = 0.01) and TIBC (*r* = −0.50, *p* = 0.01). Notably, 25(OH)D did not correlate with BMI or PTH in BD.

### 3.4. Sex-Adjusted Analysis in BD

Because the BD group comprised equal numbers of males and females (16/16), and both 25(OH)D and haemoglobin differ by sex (male median 25(OH)D 22.4 vs. female 9.7 ng/mL, *p* = 0.002; male haemoglobin 15.2 vs. female 12.2 g/dL, *p* < 0.001), partial Spearman correlations controlling for sex were performed. After sex adjustment, the 25(OH)D-haemoglobin association remained significant (partial *r* = 0.43, *p* = 0.01), as did 25(OH)D-haematocrit (partial *r* = 0.42, *p* = 0.02) and 25(OH)D-triglycerides (partial *r* = 0.43, *p* = 0.02). However, the correlations with ferritin (partial *r* = 0.20, *p* = 0.27), ESR (partial *r* = −0.27, *p* = 0.14), uric acid (partial *r* = 0.12, *p* = 0.51), and TSAT (partial *r* = 0.27, *p* = 0.20) were attenuated to non-significance after sex adjustment (Table 2). Sex-stratified analysis within BD females (*n* = 16) confirmed a strong 25(OH)D-haemoglobin correlation (*r* = 0.65, *p* = 0.006); the correlation in males (*n* = 16) was positive but did not reach significance (*r* = 0.26, *p* = 0.32, likely reflecting limited power).

### 3.5. Vitamin D Tertile Analysis

Tertile analysis confirmed disease-specific dose–response patterns (Table 3). In SLE tertiles, BMI decreased progressively from 27.2 to 22.6 kg/m^2^ (*p* = 0.01) and PTH from 67.6 to 41.9 pg/mL (*p* = 0.008) across increasing vitamin D tertiles, while haematological and inflammatory parameters did not differ.

In BD tertiles, haemoglobin increased from 11.8 to 15.3 g/dL (*p* = 0.001), ferritin from 13.7 to 71.2 ng/mL (*p* = 0.02), and uric acid from 3.9 to 4.7 mg/dL (*p* = 0.03) across increasing vitamin D tertiles. ESR decreased from 19.0 to 6.0 mm/h (*p* = 0.03). BMI and PTH did not differ across BD tertiles.

### 3.6. Vitamin D Deficiency Subgroup Analysis

When patients were dichotomised by vitamin D status (<20 vs. ≥20 ng/mL), BD patients with deficiency had significantly lower haemoglobin (13.8 vs. 15.2 g/dL, *p* = 0.006), lower ferritin (17.6 vs. 70.0 ng/mL, *p* = 0.02), and higher ESR (18.5 vs. 6.0 mm/h, *p* = 0.02). In SLE, only PTH was significantly higher in the deficient group (66.7 vs. 43.3 pg/mL, *p* = 0.01).

## 4. Discussion

This study reveals that despite comparable prevalence of vitamin D deficiency in SLE and BD (64.9% vs. 62.5%), the clinical correlates of 25(OH)D levels are strikingly disease-specific. In SLE, vitamin D shows a metabolic-skeletal pattern, correlating with BMI and PTH. In BD, vitamin D displays an entirely different profile dominated by inflammatory and haematological associations, including strong correlations with haemoglobin, iron stores, ESR, and uric acid. To our knowledge, this is the first study to directly compare vitamin D correlation profiles between SLE and BD, and the divergent patterns observed may reflect fundamental differences in pathophysiological interactions between vitamin D and each disease.

The high prevalence of vitamin D deficiency in our SLE cohort (64.9%) is consistent with the large meta-analysis by Islam et al. [3], which pooled 34 studies and found significantly lower 25(OH)D in SLE patients compared with healthy controls. Similarly, the BD deficiency rate (62.5%) aligns with previous observations in Turkish BD cohorts [4,5]. Recent Mendelian randomisation analyses by Ding et al. [17] have provided genetic evidence that SLE itself may causally reduce vitamin D levels, rather than the reverse, supporting the concept that disease-specific mechanisms drive vitamin D deficiency in SLE.

The SLE pattern, characterised by inverse correlations between 25(OH)D and BMI (*r* = −0.470) and PTH (*r* = −0.402), is consistent with the well-established metabolic consequences of vitamin D deficiency. Adipose tissue sequesters vitamin D, and BMI is a strong predictor of 25(OH)D levels in the general population [2,7]. The SLE-specific prominence of this metabolic axis, with no significant haematological or inflammatory correlates, suggests that in SLE, the relationship between vitamin D and clinical parameters may be driven primarily by body composition rather than disease-related inflammation. This finding is consistent with prior SLE studies showing that vitamin D associations with disease activity are attenuated after BMI adjustment [6,10,18]. Notably, Chew et al. [19], in a large international SLE inception cohort (*n* = 1163), demonstrated that lower 25(OH)D was associated with metabolic syndrome and insulin resistance, and that glucocorticoid exposure independently contributed to insulin resistance. Our finding that vitamin D did not correlate with HOMA-IR in SLE contrasts with these results, possibly due to our smaller sample size or the absence of disease activity adjustment.

The relationship between vitamin D and metabolic syndrome in SLE remains debated. Wang et al. [20] found that lower 25(OH)D was associated with metabolic syndrome and decreased HDL in premenopausal Chinese SLE women, whereas García-Carrasco et al. [21] reported no independent association after adjusting for body mass. Our data support the interpretation that BMI may be a primary confounder in the vitamin D-metabolic axis in SLE, as the strong vitamin D-BMI correlation (*r* = −0.470) was the dominant finding in this group.

The BD pattern is distinctly different profile and, to our knowledge, has not been previously described. The strong positive correlation between 25(OH)D and haemoglobin (*r* = 0.634, *p* < 0.001), the strongest association observed in this study, along with correlations with ferritin (*r* = 0.469), TSAT (*r* = 0.450), and inverse TIBC (*r* = −0.500), suggest the presence of a potential iron-vitamin D interaction in BD. However, because both vitamin D and haemoglobin are influenced by sex, and the BD group had equal proportions of males and females, sex confounding must be carefully considered. Partial correlations controlling for sex confirmed that the 25(OH)D-haemoglobin association remained significant (partial *r* = 0.434, *p* = 0.013), as did haematocrit and triglycerides. In contrast, the correlations with ferritin, ESR, uric acid, and TSAT were attenuated to non-significance after sex adjustment, indicating that these associations were at least partly driven by sex differences. The within-sex analysis further supported the 25(OH)D-haemoglobin link, with a strong correlation in BD females (*r* = 0.650, *p* = 0.006). The concurrent inverse correlation with ESR (*r* = −0.442), although attenuated after sex adjustment, suggests that this pattern may be partly inflammation-mediated.

In BD, systemic inflammation may simultaneously drive both vitamin D reduction and anaemia of chronic disease through hepcidin-mediated iron sequestration [8,22,23]. Vitamin D has been shown to directly suppress hepcidin transcription in hepatocytes and monocytes [23], and vitamin D deficiency may therefore exacerbate the iron-trapping effect of inflammation, potentially contributing to a vicious cycle of low vitamin D, elevated hepcidin, iron sequestration, and anaemia. The dose–response relationship across BD vitamin D tertiles, with haemoglobin increasing from 11.8 to 15.3 g/dL (*p* = 0.001), supports this interpretation, although the tertile analysis was not sex-adjusted.

The absence of vitamin D-HOMA-IR correlations in either disease group contrasts with some general population studies [7] but is consistent with the complex metabolic milieu of autoimmune diseases where corticosteroid use, chronic inflammation, and disease-specific metabolic perturbations may obscure the vitamin D-insulin sensitivity relationship [9,10,19]. Kim et al. [24] recently showed that seasonal vitamin D levels were associated with achieving lupus low disease activity state, emphasising that vitamin D effects in SLE may operate through immune pathways rather than metabolic ones.

Several mechanisms may explain the disease-specific patterns. In SLE, photosensitivity-driven sun avoidance is a major cause of vitamin D deficiency, and this cause is relatively independent of disease activity [6,18]. Nguyen et al. [18] reviewed the evidence supporting vitamin D as both a pathogenic factor and a contributor to fatigue in SLE, noting that the relationship between vitamin D and disease activity is confounded by photoprotective behaviour, corticosteroid use, and renal impairment. In BD, by contrast, photosensitivity is not a clinical feature, and the disease itself may drive vitamin D deficiency through inflammation-mediated pathways. Pro-inflammatory cytokines reduce renal 1-alpha-hydroxylase activity, increase vitamin D catabolism via CYP24A1, and simultaneously cause anaemia of chronic disease [8,22,23]. This shared inflammatory root may explain why vitamin D and haematological parameters co-vary in BD but not in SLE, where vitamin D deficiency is driven primarily by sun avoidance and adiposity. The immunomodulatory role of vitamin D in BD has been demonstrated by Hamzaoui et al. [5] and Do et al. [11], who showed that vitamin D modulates Toll-like receptor expression on monocytes in BD patients, supporting a direct anti-inflammatory mechanism.

This study has several important limitations. The sample sizes are modest (38 SLE, 32 BD), and multiple correlations were tested without multiplicity correction; accordingly, some significant associations may represent type I errors, and all findings should be interpreted as hypothesis-generating. Indeed, several BD correlations (ferritin, ESR, uric acid, TSAT) were attenuated to non-significance after sex adjustment, underscoring the importance of confounding control in small cohorts. Disease activity was not assessed with validated indices (SLEDAI, Behçet’s Disease Current Activity Form), which limits interpretation; ESR served as an indirect inflammatory marker but is not a substitute for composite disease activity scores. The sex distribution differed significantly between groups (86.8% vs. 50.0% female); although partial correlations controlling for sex were performed within the BD group, multivariate regression was not feasible given the limited sample size. Seasonal variation in vitamin D was not controlled for, which is relevant given the demonstrated seasonal effects on vitamin D in SLE [24]. However, because both disease groups were recruited from the same centre during the same study period, this effect is likely to have affected both groups similarly. Medication use, including glucocorticoids and immunosuppressive therapies, was not incorporated into the analyses and may have influenced vitamin D concentrations. Information regarding sun exposure, dietary vitamin D intake, and supplementation history was unavailable. Body composition beyond BMI (waist circumference, visceral fat) was not measured. Hepcidin, the key mediator of the proposed iron–inflammation–vitamin D axis, was not available; without hepcidin data, the mechanistic interpretation remains hypothetical. The cross-sectional design precludes causal inference.

## 5. Conclusions

Vitamin D deficiency in SLE and BD, despite equal prevalence, shows disease-specific clinical correlate profiles. In SLE, vitamin D associates with metabolic skeletal parameters (BMI, PTH), while in BD, it associates with haemoglobin independently of sex, though some iron and inflammatory correlations were partly sex-confounded. These findings suggest that the clinical significance of vitamin D deficiency may differ between autoimmune diseases and that interpretation of vitamin D status may benefit from consideration of the underlying disease context. Prospective studies with larger cohorts, disease activity measures, hepcidin levels, and multivariate adjustment are needed to confirm and extend these observations.

## Figures and Tables

**Table 1:** Patient characteristics.

	SLE (*n* = 38)	BD (*n* = 32)	*p*
Age, years	39.3 ± 12.1	40.4 ± 11.7	0.70
Female, *n* (%)	33 (86.8)	16 (50.0)	**<0.001**
BMI, kg/m^2^	24.6 (22.0–27.4)	24.1 (20.9–28.2)	0.73
Disease duration, mo	90 (51–141)	114 (57–159)	0.33
***Vitamin D and bone***			
25(OH)D, ng/mL	16.2 (10.0–25.4)	13.9 (9.4–23.8)	0.37
Deficient (<20), *n* (%)	24 (64.9)	20 (62.5)	—
Sufficient (≥30), *n* (%)	8 (21.6)	3 (9.4)	—
PTH, pg/mL	60.7 (41.2–73.2)	70.8 (43.7–83.3)	0.36
Calcium, mg/dL	9.0 (8.5–9.4)	9.2 (8.8–9.6)	0.38
ALP, U/L	60.5 (52.5–75.8)	75.0 (60.5–100.5)	**0.04**
***Iron and haematology***			
Serum iron, µg/dL	46.0 (34.0–64.0)	75.0 (44.0–94.0)	**0.02**
TIBC, µg/dL	333 (308–379)	372 (327–397)	0.18
Ferritin, ng/mL	27.3 (15.8–58.2)	31.6 (13.6–90.0)	0.75
TSAT, %	13.9 (9.8–21.5)	20.7 (11.7–32.5)	**0.048**
Haemoglobin, g/dL	12.7 ± 1.7	13.9 ± 1.9	**0.007**
***Inflammatory***			
ESR, mm/h	23 (14–40)	11.5 (6.0–22.8)	**0.03**
CRP, mg/L	3.1 (3.1–6.6)	3.1 (3.1–3.4)	0.09
Albumin, g/dL	4.2 (4.0–4.4)	4.5 (4.3–4.7)	**0.002**
***Metabolic***			
Triglycerides, mg/dL	115 (84–178)	110.5 (97–142)	0.49
HDL, mg/dL	43.4 (37.8–51.3)	47.4 (39.1–55.0)	0.48
Uric acid, mg/dL	4.2 (3.6–5.3)	4.2 (3.9–5.1)	0.69
HOMA-IR	1.9 (1.3–3.0)	1.7 (1.0–3.0)	0.37

Data are mean ± SD or median (IQR). Bold *p*-values: <0.05. TSAT: transferrin saturation; SLE: systemic lupus erythematosus; BD: Behçet’s disease; BMI: body mass index; PTH: parathyroid hormone; ALP: alkaline phosphatase; TIBC: total iron-binding capacity; ESR: erythrocyte sedimentation rate; CRP: C-reactive protein; HDL: high-density lipoprotein; HOMA-IR: homeostatic model assessment for insulin resistance.

**Table 2:** Spearman correlations between 25(OH)D and clinical parameters by disease group.

Variable	SLE *r*	*p*	BD *r*	*p*	Pattern
BMI	**−0.47**	**0.003**	0.045	0.80	SLE: metabolic
PTH	**−0.40**	**0.01**	−0.30	0.11	SLE: bone
Haemoglobin	0.07	0.67	**0.63**	**<0.001**	BD: haem
Haematocrit	0.14	0.39	**0.58**	**<0.001**	BD: haem
Ferritin	0.13	0.47	**0.47**	**0.008**	BD: iron
TIBC	−0.04	0.80	**−0.50**	**0.01**	BD: iron
TSAT	0.12	0.48	**0.45**	**0.02**	BD: iron
ESR	0.06	0.71	**−0.44**	**0.01**	BD: inflam
Uric acid	−0.10	0.54	**0.48**	**0.005**	BD: metabolic
Triglycerides	−0.16	0.35	**0.44**	**0.01**	BD: lipid
HOMA-IR	0.02	0.9	0.09	0.64	—
Fasting glucose	−0.13	0.46	0.27	0.15	—
CRP	−0.02	0.90	−0.12	0.52	—

Bold: *p* < 0.05. No multiplicity correction; interpret as exploratory. In BD, partial Spearman correlations controlling for sex confirmed significance for haemoglobin (partial *r* = 0.43, *p* = 0.01), haematocrit (partial *r* = 0.42, *p* = 0.02), and triglycerides (partial *r* = 0.43, *p* = 0.02); ferritin, ESR, uric acid, and TSAT were attenuated to non-significance after sex adjustment (see text). BMI: body mass index; PTH: parathyroid hormone; TIBC: total iron-binding capacity; TSAT: transferrin saturation; ESR: erythrocyte sedimentation rate; HOMA-IR: homeostatic model assessment for insulin resistance.

**Table 3:** Clinical parameters across disease-specific 25(OH)D tertiles.

	**SLE T1**	**SLE T2**	**SLE T3**	***p***	**BD T1**	**BD T2**	**BD T3**	***p***
25(OH)D cut	<11.5	11.5–22	>22		<9.7	9.7–20.1	>20.1	
*n*	12	13	12		11	10	11	
BMI	27.2	23.9	22.6	**0.01**	22.5	25.2	24.2	0.94
PTH	67.6	66.5	41.9	**0.008**	74.9	76.9	52.6	0.51
Haemoglobin	12.6	12.6	12.2	0.81	11.8	14.4	15.3	**0.001**
Ferritin	39.3	20.7	57.1	0.43	13.7	35.9	71.2	**0.02**
ESR	22.5	19.0	35.0	0.48	19.0	9.0	6.0	**0.03**
Uric acid	—	—	—	—	3.9	4.1	4.7	**0.03**
HOMA-IR	2.2	1.6	2.2	0.40	1.6	2.6	1.5	0.52

Median values shown (Kruskal–Wallis test). Bold: *p* < 0.05. SLE tertiles: T1 < 11.5, T2 = 11.5–22.0, T3 > 22.0 ng/mL. BD tertiles: T1 < 9.7, T2 = 9.7–20.1, T3 > 20.1 ng/mL. SLE: systemic lupus erythematosus; BD: Behçet’s disease; BMI: body mass index; PTH: parathyroid hormone; ESR: erythrocyte sedimentation rate; HOMA-IR: homeostatic model assessment for insulin resistance.

## Data Availability

The data supporting the findings of this study are not publicly available due to ethical and privacy restrictions but may be provided by the corresponding author upon reasonable request.

## Abbreviations

The following abbreviations are used in this manuscript:

25(OH)D	Serum 25-hydroxyvitamin D
SLE	Systemic lupus erythematosus
BD	Behçet’s disease
PTH	Parathyroid hormone
ALP	Alkaline phosphatase
TIBC	Total iron-binding capacity
TSAT	Transferrin saturation
HDL	High-density lipoprotein
LDL	Low-density lipoprotein
CRP	C-reactive protein
ESR	Erythrocyte sedimentation rate
GFR	Glomerular filtration rate
HOMA-IR	Homeostatic model assessment of insulin resistance
BMI	Body mass index
